# Epigenetic memory as crucial contributing factor in directing the differentiation of human iPSC into pancreatic β-cells in vitro

**DOI:** 10.1007/s00441-025-03952-8

**Published:** 2025-01-30

**Authors:** Abdoulaye Diane, Razik Bin Abdul Mu-U-Min, Heba Hussain Al-Siddiqi

**Affiliations:** Diabetes Research Center, Qatar Biomedical Research Institute (QBRI), Qatar Foundation (QF), Hamad Bin Khalifa University (HBKU), Doha, Qatar

**Keywords:** Epigenetic, Reprogramming, Differentiation, IPSC-derived β-cells, Diabetes mellitus

## Abstract

Impaired insulin secretion contributes to the pathogenesis of type 1 diabetes mellitus through autoimmune destruction of pancreatic β-cells and the pathogenesis of severe forms of type 2 diabetes mellitus through β-cell dedifferentiation and other mechanisms. Replenishment of malfunctioning β-cells via islet transplantation has the potential to induce long-term glycemic control in the body. However, this treatment option cannot widely be implemented in clinical due to healthy islet donor shortage. Emerging β-cell replacement with human-induced pluripotent stem cell (iPSC) provides high remedial therapy hopes. Thus, tremendous progress has been made in developing β-cell differentiation protocols in vitro; however, most of the differentiated iPSC-derived β-cells showed immature phenotypes associated with low efficiency depending on the iPSC lines used, creating a crucial barrier for their clinical implementation. Multiple mechanisms including differences in genetic, cell cycle patterns, and mitochondrial dysfunction underlie the defective differentiation propensity of iPSC into insulin-producing β-cells. Accumulating evidence recently indicated that, following the reprogramming, epigenetic memory inherited from parental cells substantially affects the differentiation capacity of many iPSC lines. Therefore, differences in epigenetic signature are likely to be essential contributing factors influencing the propensity of iPSC differentiation. In this review, we will document the impact of the epigenome on the reprogramming efficacy and differentiation potential of iPSCs and how targeting the epigenetic residual memory could be an additional strategy to improve the differentiation efficiency of existing protocols to generate fully functional hPSC-derived pancreatic β-cells for diabetes therapy and drug screening.

## Introduction

Diabetes mellitus (DM) is a chronic and complex metabolic disorder that results from insulin-secreting β-cell loss or dysfunction (Chawla et al. 2016). The recent report of the International Diabetes Federation (IDF) estimated that 537 million adults worldwide are living with some form of diabetes (www.idf.org), with type 1 diabetes (T1DM) accounting ~ 10% of reported diabetic patients. Type 2 diabetes (T2DM), the most common, affecting > 90% of people diagnosed with DM, results from an inability of pancreatic β-cells to produce adequate insulin to stimulate glucose utilization by peripheral metabolically active organs to maintain glucose homeostasis (Lee et al. 2022). Now, it is well acknowledged that both T1DM and severe forms of T2DM commonly share a dysfunction of the pancreatic β-cells that negatively impacts insulin secretion. While modern diabetes therapy based on exogenous insulin injection is considered a life-saving treatment for managing the disease, it is unfortunately associated with acute hypoglycemia episodes and weight gain for many patients (Sun et al. 2021b) and not as efficient as the endogenous pancreatic islets for glycemic control. Transplantation of cadaveric islets or surrogate insulin-producing β-cells from human pluripotent stem cells is an effective alternative therapy to restore normoglycemia when endogenous β-cells have already been practically depleted (Ramzy et al. 2021; Shapiro et al. 2000; Sun et al. 2021b). Human pluripotent stem cells (hPSC), including embryonic stem cells (ESC) and induced pluripotent stem cells (iPSC), could be differentiated into any cell type of somatic cells in the body and considered to be popular and valuable alternative source to cadaveric islets because of their infinite self-renewal competency. Thus, hPSC-derived pancreatic pseudoislets also known as autologous iPSC-derived β cells potentially offer an alternative inexhaustible source of insulin-producing β-cells for potential diabetes therapy in the future. In T2DM condition, autologous iPSC-derived β-cells would overcome allogeneic immune rejection as compared to embryonic stem cell-derived β-cells. Nevertheless, in the case of T1DM, the use of encapsulation device still remains crucial to prevent autoimmune attacks against the transplanted patient’s own iPSC-derived β-cells to ensure graft survival. Over the last two decades, great effort has been concentrated on developing in vitro protocols to reproducibly differentiate hPSCs into insulin-producing cells with key features of bona fide mature β-like cells using multi-stages directed differentiation protocols that recapitulate the specific stages of pancreas development (Hogrebe et al. 2020; Iworima et al. 2024; Veres et al. 2019). However, despite recent advances in developing β-cell differentiation protocols in vitro, most of the differentiated hPSC-derived insulin-expressing β-cells are functionally immature. Typical low or absence expression of key transcription factors as mature β-cell markers includes MAFA, NEUROD1, NKX6.1, PDX1, SIX2, and UCN3. Also, β-cell maturity is determined by the absence of “disallowed” genes that interfere with β-cell function including Ldha, Mct1, SLC16A1, Hk1, and Hk2 (Blum et al. 2012; Lemaire et al. 2016; Pagliuca et al. 2014; Zhu et al. 2017). One prominent characteristic of mature β-cells is glucose-stimulated insulin secretion (GSIS). IThe immature phenotype in generated iPSC-derived β-cells in vitro is also characterized by the absence or low amplitude of GSIS as compared to native pancreatic β-cells (Diane et al. 2022). The presence of polyhormonal cells is also another sign of immature phenotype. Indeed, there is a substantial inconsistency in the differentiation efficiency depending on the hPSC lines used with some lines producing low efficiency. This in vitro low differentiation efficiency in hPSC-derived β-cell generation creates a crucial barrier for clinical implementation. Multiple mechanisms including differences in genetic and cell cycle patterns underlie defective differentiation propensity of hPSC into insulin-producing β-cells (Bock et al. 2011; Chetty et al. 2013; Li et al. 2019). iPSCs exhibit higher intrinsic genetic heterogeneity as compared to ESCs. Genome instability refers to a range of DNA alterations, from deletions, insertions, and point mutations to chromosomal rearrangements. As the genome of iPSC closely reproduces that of the somatic cells of origin, they may possess genetic abnormalities, which would compromise their differentiation efficiency and safety. Thus, mutations could be present in parental somatic cells and then transferred during reprogramming or acquired through iPSC generation or during extended passaging and prolonged culturing. Taken together, pre-existing or de novo mutations could greatly compromise the genetic stability of iPSC and subsequently affect the differentiation efficiency of iPSC-derived β-cells. Additionally, substantial findings support a critical role for cell cycle machinery in cell fate decision (Pauklin and Vallier 2013). The generation of adult β-cells mostly takes place through self-replication; therefore, it has been shown that the cell cycle plays a fundamental role in iPSC-derived β-cell differentiation (El-Badawy and El-Badri 2016). β-cells are well-known to be among the most slowly replicating cells in the body; based on their unique cell cycle machinery, a recent report suggested that special modulation of that molecular pathways controlling the cell cycle may be necessary to achieve effective differentiation of iPSC into β cells. Key cell cycle regulatory proteins such as Cdk4 and its binding partner, cyclin D2, are demonstrated to be critical in β-cell development (Fujii-Yamamoto et al. 2005; Neganova et al. 2009). Cdk4 promotes β cell expansion via activation of PDX1 and NGN3 expression (Kim and Rane 2011). Additionally, Cdk4–cyclin D2 overexpression leads to active proliferation of pancreatic progenitors present within the islets rather than self-replication of pre-existing β-cells (Chen et al. 2012). Moreover, Cdk4–cyclin D2 overexpression generated functional β-cells within a short time period in vitro (El-Badawy and El-Badri 2016). Accumulating evidence recently indicated that, following the reprogramming, epigenetic memory inherited from parental cells substantially impacts the differentiation capacity of the resulted iPSC lines (Kim et al. 2011). In this review, we will document the impact of the epigenetic signaling mechanism on the reprogramming and differentiation potential of hPSCs and how residual epigenetic memory modulation could be an additional strategy during in vitro differentiation protocols to generate functional hPSC-derived pancreatic β-cells from multiple donors for diabetes therapy and drug screening.

## Fundamental concepts of epigenetics in human disease etiology

“Epigenetic” is a term referring to heritable changes in gene function that take place in the cells by mechanisms different from changes in DNA sequence. It is a well-established mechanism that controls the expression of genes in addition to the canonical transcriptional and translational pathways. Epigenetic regulation plays an important role in both animal and plant development and is required to achieve stable repression of genes in specific cell types as well as at defined developmental stage. Today, the three major recognized and well-characterized epigenomes include the following: DNA methylation, histone modifications (e.g., acetylation), and non-coding RNA-associated gene silencing. DNA methylation occurs on a cytosine, mainly in CG context (so-called CpG sites). Methyltransferases, i.e., DNA (cytosine-5)-methyltransferase 1 (DNMT1), and DNMT3A/B are responsible for adding methyl groups to the DNA during replication and/or de novo methylation by using S-adenosyl-L-methionine as a methyl donor. Besides DNA methylation, hundreds of post-translational modifications such as acetylation and ubiquitylation have been found on amino terminus or tails of histones, and several enzymes are responsible for fixing and removing histone modifications, contributing to a complex epigenetic regulation (Zhang et al. 2015). Short-silencing non-coding RNAs such as miRNAs have recently emerged as key players in the regulation of the cell phenotype by altering the accessibility of genes to the transcriptional machinery. They repress or activate the expression of gene transcripts and conversely; these non-coding RNAs can themselves be epigenetically regulated (Alhazzaa et al. 2023). Different kinds of uterine environmental stressors, including maternal dietary modifications and assisted reproductive technology (e.g., intrauterine insemination) may perturb the maintenance of epigenetic marks and can thus heritably affect gene expression of the offspring (Arnaud and Feil 2005; Khosla et al. 2001). Substantial evidence has demonstrated that epigenetic regulation has important effects on embryogenesis and development and causally involved in many metabolic diseases including obesity and diabetes (Bansal and Pinney 2017; Delaval et al. 2006; Ling and Ronn 2019; Wu et al. 2023).

## Role of epigenetics in diabetes mellitus

DNA methylation and histone modifications (e.g., acetylation, phosphorylation, ribosylation, and ubiquitylation) (Zhang et al. 2020) are the two main known mechanisms by which epigenetics affects cell phenotype and subsequently many biological processes. The role of epigenetics in the development of metabolic syndrome including DM was first described by David Barker and colleagues in their developmental origins of health and disease (DOHaD) hypothesis, showing a strong relationship between low birth weight and the increased risk of T2DM in later life in Hertfordshire population in England (Hales and Barker 2013). Following this observation, animal models of fetal programming have been developed to investigate the underlying mechanisms. There was mounting evidence from many animal models that intra-uterine growth restriction (IUGR) has profound negative effects on the expression of genes controlling glucose and energy metabolism in pancreatic islets (Woo and Patti 2008). DNA methylation was reported to be one potential epigenetic mechanism by which an IUGR (i.e., in-utero insult) can lead to the development of diabetes in adult offspring (Drake and Walker 2004). Epigenetic modifications have been shown to play a critical role in regulating the expression of key genes involved in pancreas development and function (Kaimala et al. 2022). A maternal low-protein diet has been shown to reduce the expression of Hnf4α, a pancreatic transcription factor in offspring. This reduction was associated with increased DNA methylation, along with increased silencing histone modifications, and reduced activating histone modifications (Sandovici et al. 2011). Similarly, pancreatic homeobox domain 1 (Pdx1), a key pancreatic transcription factor regulating pancreatic differentiation, has been demonstrated to have reduced expression in IUGR β-cells (Stoffers et al. 2003) associated with epigenetic changes including an increase in DNA methylation and silencing histone modification at CpG sites within the Pdx1 promoter as well as in the insulin gene (Park et al. 2008; Yang et al. 2011). Additionally, a genome-wide DNA methylation profile study using the Infinium27K array reported increased methylated CpG sites in islets of T2DM versus non-diabetic subjects (Volkmar et al. 2012). These findings identified DNA methylation in the islets as a potential contributing factor to the pathogenesis of T2DM. Pertaining to T1DM, many studies have reported significant changes in DNA methylation at CpG sites in immune cells including peripheral lymphocytes (Stefan et al. 2014) and monocytes (Rakyan et al. 2011). Moreover, study in lymphoblast cell lines from 3 pairs of monozygotic twins discordant for T1DM and 6 pairs of monozygotic twins concordant for T1DM found increased DNA methylation in genes involved in the immune response pathways that had previously been associated with T1DM pathogenesis (Stefan et al. 2014), pointing out DNA methylation as a strong epigenetic factor on T1DM etiology. These studies collectively indicated that both T1DM and T2DM commonly shared epigenetic alterations as one of the underlying molecular mechanisms in their pathogenesis. As mentioned above and elsewhere (Sun et al. 2021a; Volkov et al. 2017), epigenetic modifications play a critical role in establishing and maintaining the identity and function of β-cells.

**Fig. 1:**
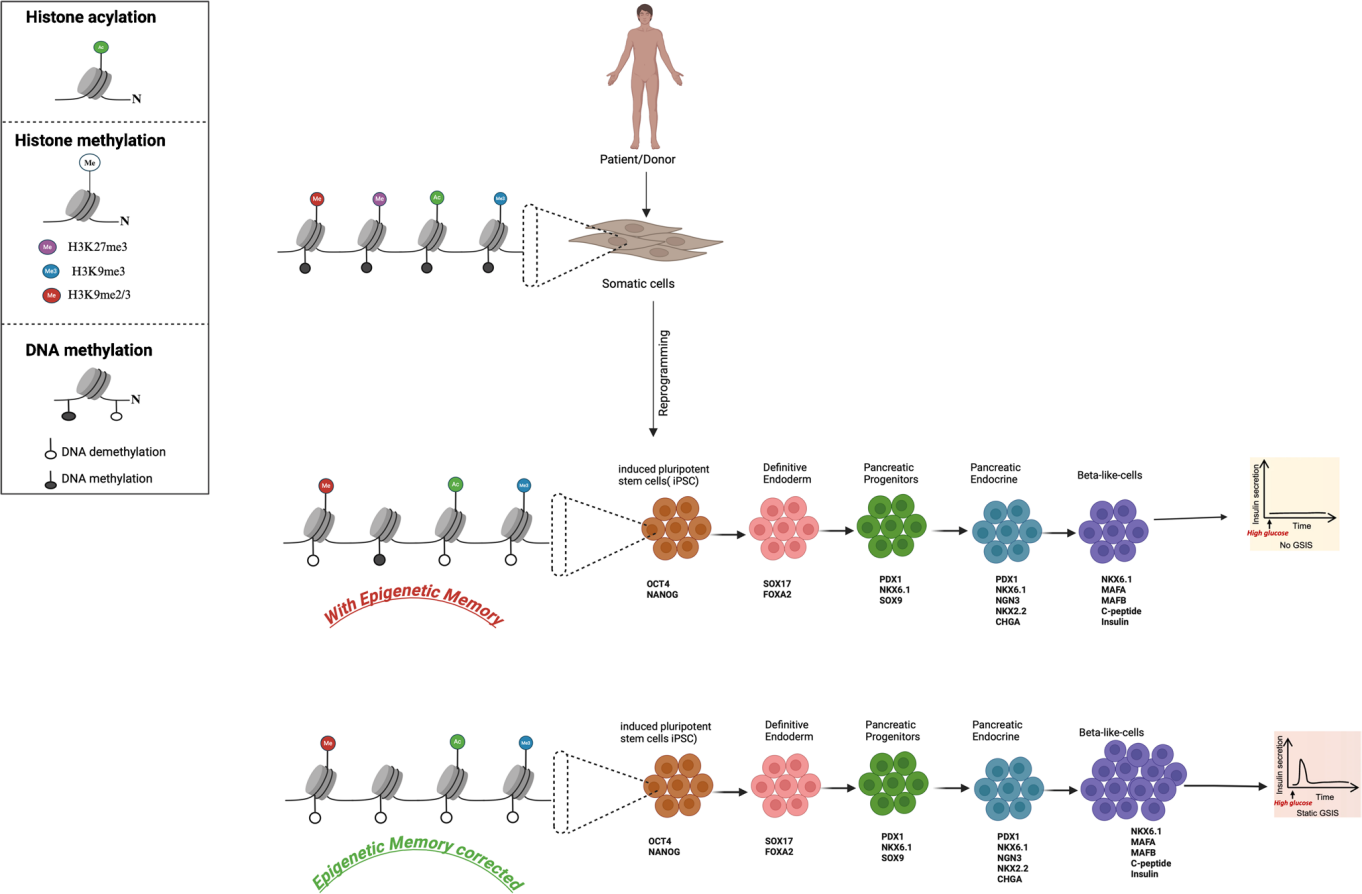
Schematic of the stepwise differentiation protocol for the generation of iPSC-derived β-cells and the potential impact of epigenetic memory on the differentiation efficiency and functionality. Profound changes in the epigenetic landscape occur during the reprogramming process of somatic cells into iPSCs such as histone H3 acetylation and histone H3 methylation along with DNA modification (i.e., DNA methylation or DNA demethylation). Unsuccessful reprogramming of somatic cells can lead to the generation of iPSC with residual epigenetic memory that could substantially impact the β-cell lineage differentiation potential. Epigenetic memory correction using either pharmacological agents or gene editing tools may offer an important and unique opportunity to improve the efficiency and reproducibility of the generation of fully functional iPSC-derived pancreatic β-cells from multiple donors. Illustration created with BioRender.com

Differentiation from a stem cell into a fully somatic cell is achieved and maintained through epigenetic regulation of gene expression, consisting of different mechanisms including DNA methylation, histone modification, packaging and rearrangement of nucleosome, and strict order of chromatin structures (Cai et al. 2012). Consequently, the complete reversal of this complex process requires extensive reprogramming that makes it susceptible to errors and/or inefficient (Plath and Lowry 2011), such as the direct reprogramming of somatic cells from adult cells to induced pluripotent stem cells (iPSC). This is achieved through ectopic expression of Yamanaka’s four defined transcription factors: Oct4, Sox2, Klf4, and c-Myc (OSKM). Like embryonic stem cells (ESC), induced pluripotent stem cells (iPSC) can be differentiated into desired cell lineages, including β-cells for disease modeling and potential therapeutic applications. However, many barriers exist such as retention of epigenetic memory of the iPSC inherited from parental somatic cells of origin that may influence the differentiation propensity of the iPSC lines (Fig. 1).

## Epigenetic memory in iPSCs impacts the differentiation efficiency toward β-cells

Over the recent years, cell therapy for chronic metabolic diseases such as DM has become a hot topic. The basic principle of the stem cell-based approach in diabetes therapy following cadaveric islet transplantation using Edmonton protocol (Shapiro et al. 2000) is to differentiate hPSCs into insulin-expressing β-cells and transplant them to diabetic patients which helps to circumvent healthy islet donor shortage. Several types of stem cells have been used for differentiation into β-cells. However, the choice of stem cell type for differentiation is critical; this is because different stem cell types are associated with different advantages and disadvantages (Stonesifer et al. 2017). For example, ESCs exhibit high proliferative and pluripotent potential but are associated with ethical concerns and the risk of immune rejection (Matsumoto and Shimoda 2020). As for mesenchymal stem cells (MSCs), they exhibit reduced immunogenicity and tumorigenicity but demonstrate a limited capacity to differentiate into β-cells (Thanaskody et al. 2022). Of these various stem cell types, induced pluripotent stem cells (iPSCs) are currently regarded as highly promising for cell therapy for diabetes. Furthermore, patient-specific PSC-derived β-cells circumvent the ethical concerns that are often associated with ESCs (Maxwell and Millman 2021). However, despite sharing all key characteristics with ESCs, iPSCs generated by reprogramming somatic cells still face many challenges, such as epigenetic memory. Epigenetic memory refers to the retention of the characteristics of the original somatic cells at the epigenetic level by iPSCs; this memory may result in the limitation or preference of iPSCs differentiation into β-cells.

The reprogramming process is known to considerably alter the epigenetic and chromatin landscapes of the cells by resetting DNA methylation, alteration of histone modifications, and restructuration of the chromatin. These changes in the epigenome are crucial as they wipe off the original cellular identity and simultaneously activate and repress genes related to pluripotency and differentiation, respectively (Godini et al. 2018). However, epigenetic reprogramming is not always 100% efficient and yields sometimes incomplete reprogrammed cells resulting in residual epigenetic memory in generated iPSCs (Fig. 1). In practice, it has been demonstrated that even rigorously selected iPSCs retain epigenetic mark characteristic of the donor cell (Kim et al. 2010). This explains the epigenetic differences between iPSCs and ESCs but also within different iPSC lines. The epigenetic variations within iPSCs may cause heterogeneity in the pluripotency and subsequently their differentiation propensity. More importantly, reports have demonstrated that cellular origin and the age of the donor’s cells limit the efficiency and fidelity of reprogramming. Specifically, aged cells exhibit higher levels of Ink4/Arf locus that subsequently decrease their reprogramming efficiency. Conversely, lacking the INK4/ARF locus or silencing INK4a or ARF alone improves reprogramming efficiency (Li et al. 2009). Moreover, Eminli et al. have reported that the stages of development determine the reprogramming potential of somatic cells into iPSC. They found that reprogramming efficiency is lower in terminally differentiated blood cells than blood progenitors (Eminli et al. 2009). Collectively, these findings indicate that the reprogramming efficiency of somatic cells into iPSC declines with increasing age and differentiation status of the donor cell but also varies with the methylation state of the donor nucleus (Blelloch et al. 2006; Hu et al. 2022). Besides age, the susceptibility to reprogramming depends also on the cellular origin. Interestingly, it has been demonstrated that donor cells from the endodermal origin, such as those from the liver, gallbladder, intestine, and cystic duct, are likely more readily reprogrammed to become β-cells compared to cells derived from non-endodermal origin (fibroblasts from the mesoderm and keratinocytes from the ectoderm), due to their inherent transcriptional and epigenetic profile, which is more developmentally and closely aligned with the pancreatic lineage. For example, cells derived from the human liver might retain certain epigenetic markers that make them more receptive to differentiation cues toward β-cells. Thus, hepatic progenitor cells were reported to transdifferentiate into β-cells in the absence of glucocorticoids and upon exposure to high glucose (Li et al. 2012). Conversely, donor cells with a highly differentiated or tightly repressed epigenetic profile such as fibroblasts showed reduced efficiency in iPSC generation and β-cell differentiation (Nakagawa et al. 2008). Moreover, keratinocytes from the ectoderm lineage reprogram more quickly than fibroblasts, indicating that the source of the tissue influences the reprogramming efficiency (Aoi et al. 2008; Maherali et al. 2008; Phetfong et al. 2016, Scesa et al. 2021). These cell-of-origin–specific differences in the reprogramming efficiency may result in the degree of epigenetic signatures (Nishino et al. 2021), indicating that iPSCs retain a transcriptional memory of their somatic cell of origin. Due to a close cooperation between the epigenomes and the transcriptomes in regulating cell growth and differentiation, epigenetic memory in iPSC would potentially team up with transcription factors to likely impact the differentiation efficiency and the functionality of iPSc-derived β-cells. Systematic reviews have all shown that by imitating the in vivo microenvironment and using 3D culture, iPSC-derived β-cells showed better efficacy and functionality than the other methods of differentiation (Kalra et al. 2018; Luo et al. 2024; Nemati et al. 2021). Recently, comprehensive genome-wide analysis and genetic and pharmacological manipulation studies that target specific epigenetic mechanisms have underscored the important role of epigenetic regulation in DNA methylation in pancreatic endocrine development and islet maturation (van Arensbergen et al. 2013, 2010). Consistently, an in vitro study on the differentiation of iPSCs into pancreatic β-cells revealed that hyper- and hypo-methylation of genomic regions coincides with the alteration of transcriptional activity of the nearby genes that induce stage-specific transcription factor expression during different stages of differentiation (Dhawan et al. 2015). Additionally, histone H3 trimethylation at lysine 27 (H3K27me3) is associated with low transcriptional activity. Disallowed genes such as lactate dehydrogenase (Ldha) and the lactate/pyruvate transporter Slc16a1 (or Mct1) known to inhibit GSIS showed low expression in mature pancreatic islets. Although gene-body methylation is mainly observed in active regions of genes, the methylation in the promoter region is reported to be more repressive (Ball et al. 2009). Thus, several reports have now established the involvement of H3K27me3 in the repression of these disallowed metabolic genes in mature β cells as evidenced by high H3K27me3 content in their promoter regions (Thorrez et al. 2011). Indeed, the low *Mct1* and *Ldha* mRNA levels in mature islets correlate to epigenetic silencing; a phenomenon normally expected to occur postnatally in humans when β-cells mature. Post-translational histone modifications (e.g., acetylation) involving histone deacetylases (HDACs) are also known to epigenetically regulate gene expression and subsequently cell fate decisions (Montgomery et al. 2007). Many pharmacological agents that amend the epigenetic landscape are being explored to enhance stem cell differentiation. These drugs target specific epigenetic mechanisms, such as DNA methylation and histone modifications, to modulate gene expression that potentially improves the efficiency of differentiation, maturation, and functionality of stem cell-derived β-cells. Thus, inhibition of HDACs was successfully used to demonstrate the roles of HDACs in pancreatic differentiation. Importantly, the maintenance of acetylation in embryonic pancreas explants with trichostatin A and sodium butyrate (small-molecule HDAC inhibitors) promoted the pool of insulin + cells (Haumaitre et al. 2008). Moreover, romidepsin and valproic acid that class 1 and II HDACs inhibitors (Snykers et al. 2007) promote histone acetylation, which increases the expression of pancreatic transcription factors; highlighting the role of HDACs as an epigenetic factor in pancreatic β cell development. Consequently, targetting the epigenome during differentiation may help to improve the efficiency and functionality of iPSC-derived β-cell in vitro. Therefore, DNA demethylation known to play a major role in lineage specification (Wu and Zhang 2010) has been demonstrated to promote efficiently insulin-producing cell generation. More specifically, a brief exposure to cytidine analog (5-azacytidine, 1 µM for 18 h), a DNA methyltransferase inhibitor drug, and then immediately followed by differentiation sufficiently allowed the conversion of adult human skin fibroblasts into insulin-producing cells (Pennarossa et al. 2013). Hypomethylation of genes like Pdx1, NeuroD1, and MafA can improve the differentiation efficiency of iPSC-derived islets (Pennarossa et al. 2013) Similarly, Wang et al. (2019) reported that pretreatment of T1DM patient-derived iPSCs with 5-azacytidine markedly increased the efficiency of β-cell differentiation. Given the non-specific nature of current epigenetic drugs, there is a potential risk of interference with tumor suppressor genes leading to their inactivation or activation of oncogenes that may promote tumorigenesis. Caution of gene-specific methylation-modifying drug to improve the differentiation efficiency of iPSC-derived β-cells is warranted. Moreover, promoter hypomethylation has usually been shown to result in high expression of the regulated genes (Maeder et al. 2013; Sailani et al. 2019). For example, DNA hypomethylation of the imprinting control region 2 (ICR2) has been proposed as the underlying molecular mechanism of excessive β-cell mass in Beckwith-Wiedemann syndrome (Kalish et al. 2016). This observation implies that epigenetic manipulation of ICR2 may have the potential to modulate β-cell mass expansion (Wang and Pei 2019). Therefore, to test this hypothesis, Ou et al. used a TALE-TET1 fusion protein method to target and demethylate ICR2. They found that TALE-TET1-induced hypomethylation promoted mature β-cell expansion (Ou et al. 2019). More importantly, transplantation of these epigenetically edited islets into diabetic mice reversed hyperglycemia. Another epigenetic modifying tool is CRISPR-Cas9 technology. However, one of the primary concerns remains its potential to introduce unintended genetic changes, known as off-target effects (Tong et al. 2019), which could lead to significant safety issue (inhibition of tumor suppressor genes or activation of oncogenes). To reduce the likelihood of off-target effects, strategy has been developed to increase the specificity of the CRISPR-based gene editing such as a combination of gRNA and high-fidelity Cas9 variants (Doench et al. 2016), or use of multiple gRNAs to target the same gene (Zhang et al. 2021). Together, these findings further support the need to target the epigenome of iPSCs during directed differentiation protocols to efficiently generate iPSC-derived β-cells for diabetes therapy.

## Conclusion

The potential of human iPSCs to differentiate into islets makes them promising for personalized cell replacement therapy for diabetes. Although human iPSC-derived β-cells help to overcome allogenic immune rejection as compared to human ESCs, they may possess residual epigenetic memory inherited from parental cells during the reprogramming that could impact their differentiation capacity. Therefore, emerging evidence has demonstrated that differences in epigenetic signature are essential contributing factors influencing negatively the propensity of iPSCs differentiation into β-cells. This limitation highlights the need for further optimization of differentiation protocols to target epigenetic residual memory as an additional strategy during the in vitro direct differentiation to improve the efficiency of the generation of fully mature and functional iPSC-derived pancreatic β-cells. The main cause of rejection in allogeneic transplantation is T-cell recognition of human leukocyte antigens (HLA). Thus, one approach to eliminate the T-cell-mediated adaptive immune response against allogeneic material is to remove HLAs from transplanted iPSC-derived β-cells to avoid the host T-cell activation. Thus, for standardized therapeutic products, the strategy would be the use of a CRISPR/Cas9 system to selectively remove HLA in the hPSC line with higher differentiation efficiency. This strategy can be developed to establish iPSC line banks lacking HLA with low batch-to-batch variation in differentiation efficiency to match the majority of the T1DM population.

When fully functional iPSC-derived β-cells or islets are finally produced, another question and challenge remains how to safely transplant and maintain their viability and function for a long-term period in recipient patients. The challenges faced by therapies from iPSC-derived β cells and current research to tackle them are listed below:

- Islet cell lost after transplantation: often associated with poor vascularization as native islets that are highly vascularized within the pancreas to receive the proper nutrients and oxygen supply. Transplantation of iPSC-derived islets into mice has demonstrated significant cell death shortly after transplantation due to the synergistic effects of nutrient deprivation and hypoxia. Ensuring quick islet revascularization following transplantation will likely benefit β-cell survival and functionality. Therefore, in vitro co-culturing of pancreatic islet cells with human umbilical vein endothelial cells or mesenchymal stem cells is shown to protect islet cells from cell death by promoting angiogenesis in transplanted grafts (Lebreton et al. 2019; Rawal et al. 2017).
- Risk of tumorigenesis: The capacity of stem cells to self-renew and differentiate into any desired cell type underlies the risk of developing tumors. Therefore, several methods and tools have been developed for guaranteeing safety in their therapeutic applications. First, tumorigenesis can be prevented by transplanting only differentiated cells following optimized differentiation protocols. Second, approaches have been developed for the elimination of remaining undifferentiated iPSC in vitro, such as the use of chemical inhibitors (Ben-David et al. 2013), or genetic methods by introduction of suicide genes in the iPSC genome (Li and Xiang 2013).
- Graft immune rejection: Another major barrier for the clinical application of iPSC-derived β-cells is immune rejection of the graft due to human leukocyte antigen (HLA)-mismatching. T1DM patients with islet transplants currently depend on immune-suppressive drugs for preventing allograft immunity. These drugs can have side effects. Strategies to prevent allograft rejection could be prevented if iPSC-derived β-cells transplanted perfectly match with the patient HLA.
- The emergence of immune protection by encapsulation devices: to avoid graft rejection post-transplantation by bypassing the need for an immunosuppressive drugs regimen, cell encapsulation has been developed. Transplanted cells are encased by semi-permeable biocompatible material that blocks immune cells’ entry while being permeable to oxygen, nutrients, and metabolites.

In conclusion, compared to pharmacological therapy, iPSC-based therapy for diabetes is extremely complex and poses major safety, quality controls, and logistical challenges. All these factors need to be addressed for the iPSC-derived β-cell-based therapy to become widely a therapeutic reality in clinics for diabetic patients.

## Data Availability

No datasets were generated or analysed during the current study.
